# Serine Deficiency Exacerbates Inflammation and Oxidative Stress via Microbiota-Gut-Brain Axis in D-Galactose-Induced Aging Mice

**DOI:** 10.1155/2020/5821428

**Published:** 2020-03-02

**Authors:** Fengen Wang, Hongbin Zhou, Ligang Deng, Lei Wang, Jingqing Chen, Xihong Zhou

**Affiliations:** ^1^Institute of Quality Standard & Testing Technology for Agro-Products, Shandong Academy of Agricultural Sciences, Jinan 250100, China; ^2^Dalian Chengsan Husbandry Co., Ltd., Dalian, China; ^3^State Key Laboratory of Animal Nutrition, China Agricultural University, Beijing 100193, China; ^4^Key Laboratory of Agro-ecological Processes in Subtropical Region, Institute of Subtropical Agriculture, Chinese Academy of Sciences, Changsha 410125, China

## Abstract

Inflammation and oxidative stress play key roles in the process of aging and age-related diseases. Since serine availability plays important roles in the support of antioxidant and anti-inflammatory defense system, we explored whether serine deficiency affects inflammatory and oxidative status in D-galactose-induced aging mice. Male mice were randomly assigned into four groups: mice fed a basal diet, mice fed a serine- and glycine-deficient (SGD) diet, mice injected with D-galactose and fed a basal diet, and mice injected with D-galactose and fed an SGD diet. The results showed that D-galactose resulted in oxidative and inflammatory responses, while serine deficiency alone showed no such effects. However, serine deficiency significantly exacerbated oxidative stress and inflammation in D-galactose-treated mice. The composition of fecal microbiota was affected by D-galactose injection, which was characterized by decreased microbiota diversity and downregulated ratio of *Firmicutes*/*Bacteroidetes*, as well as decreased proportion of *Clostridium XIVa*. Furthermore, serine deficiency exacerbated these changes. Additionally, serine deficiency in combination with D-galactose injection significantly decreased fecal butyric acid content and gene expression of short-chain fatty acid transporters (*Slc16a3* and *Slc16a7*) and receptor (*Gpr109a*) in the brain. Finally, serine deficiency exacerbated the decrease of expression of phosphorylated AMPK and the increase of expression of phosphorylated NF*κ*B p65, which were caused by D-galactose injection. In conclusion, our results suggested that serine deficiency exacerbated inflammation and oxidative stress in D-galactose-induced aging mice. The involved mechanisms might be partially attributed to the changes in the microbiota-gut-brain axis affected by serine deficiency.

## 1. Introduction

The population of aging people is increasing rapidly worldwide, and aging is considered as being the major factor for the development of age-related diseases. Consequently, revealing the mechanisms involved in age-related diseases is critically important. Free radical theory is one of the accepted mechanisms by researchers, as increased reactive oxygen species are accumulated in the brains and they attack many kinds of biomolecules during the process of age-related diseases [1]. Additionally, inflammation has been considered to contribute to the pathogenesis of age-related disorders, and the condition often referred to as “inflammaging” [2]. Recently, increasing evidences indicated that the changes in microbiota composition have been associated with oxidative and inflammatory processes in the aging brain, as there are bidirectional communications between the brain and the gut microbiota, termed the microbiota-gut-brain axis, which enables gut microbes to communicate with the brain [3, 4]. However, studies are still needed to elucidate how the microbiota-gut-brain axis works in these processes.

Generally, diets were considered to play important roles in brain aging through the modulation of gene and protein expression [5]. Recent researchers suggested that diet-induced changes of gut microbiota also affected age-related pathogenesis [6]. Serine is a nutritionally nonessential amino acid, but it is a major substrate for the synthesis of glutathione. Recent studies demonstrated that dietary supplementation with serine showed strong anti-inflammatory and antioxidative abilities in certain mouse models [7–10]. Moreover, serine exerted beneficial effects on microbiota composition and alleviated inflammatory responses in a dextran sulfate sodium-induced colitis model in mice [11]. Importantly, the offspring mice from dams fed a serine- and glycine-deficient diet were vulnerable to oxidative stress [12]. Consequently, we conducted the present study to explore whether serine deficiency affects inflammatory and oxidative status in the brain in an aging mouse model induced by chronic D-galactose treatment, which had a similar characteristic as natural aging rodents did. Furthermore, whether and how the microbiota-gut-brain axis is involved in these effects were also explored.

## 2. Materials and Methods

### 2.1. Animals and Experimental Design

Thirty-two male C57BL/6J mice at the age of 9 weeks were purchased from SLAC Laboratory Animal Central (Changsha, China). The mice were maintained in plastic cages under standard conditions and were free to access feed and water. After an acclimatization of 2 weeks, all animals were randomly assigned into 4 groups (*n* = 8): Control group, mice were fed a basal diet and injected with saline; SGD group, mice were fed a serine- and glycine-deficient (SGD) diet and injected with saline; D-gal group, mice were fed a basal diet and injected with D-galactose; and D-SGD group, mice were fed a serine- and glycine-deficient diet and injected with D-galactose. Mice were subcutaneously injected with 1.2 g per kg body weight/day D-galactose (D-galactose solution, 0.15 g/mL). The basal diet and the serine- and glycine-deficient diet were purchased from Research Diets (New Brunswick, NJ, USA). The experiment lasted two months. The experimental protocol was approved by the Protocol Management and Review Committee of Institute of Subtropical Agriculture, and mice were treated according to the animal care guidelines of the Institute of Subtropical Agriculture (Changsha, China). At the end of the experiments, fresh feces were collected for the analysis of microbiota composition and short-chain fatty acid (SCFA) concentrations. After the mice were sacrificed, the blood samples were collected and serum were separated and stored at -20°C until analysis. The brain was also collected and immediately frozen in liquid nitrogen and stored at -80°C until analysis.

### 2.2. Biochemical Assays

Concentrations of advanced glycation end products (AGEs), malondialdehyde (MDA), tumor necrosis factor (TNF-*α*), interleukin 1*β* (IL-1*β*), and IL-6, as well as activities of superoxide dismutase (SOD), glutathione peroxidase (GSH-Px), catalase (CAT), and glutathione content were measured using commercially available kits (Meimian, Jiangsu Yutong Biological Technology Co., Ltd, Nanjing, China) based on absorption according to the manufacturer's instructions.

### 2.3. qRT-PCR

Total RNA was isolated from whole brain tissue using the TRIZOL reagents (Invitrogen, Shanghai, China). The RNA was reverse transcribed into cDNA using a cDNA Reverse Transcription Kit (Takara, Dalian, China) according to the manufacturer's instructions. RT-qPCR was performed with SYBR Green mix (Takara) as previously described [13]. The primers used to amplify the mRNA are listed in Supplementary [Supplementary-material supplementary-material-1].

### 2.4. Microbiota Profiling

DNA was extracted from fecal samples using the QIAamp DNA Stool Mini Kit (Qiagen) as previously described [14]. Bacterial 16S rRNA gene sequences (V3–V4 region) were amplified, and then, PCR were performed in a total volume of 50 mL consisting of 12.5 mL of Phusion High-Fidelity PCR Master Mix (New England BioLabs Inc., Beverly, MA, United States), 1 mL of each primer, 50 ng of template DNA, and PCR-grade water. The purified 400–450 bp PCR products were used for MiSeq Illumina sequencing performed using the Illumina HiSeq 2500 platform (Illumina Inc., San Diego, CA, United States). Followingly, the obtained paired-end reads were merged and then assigned to each sample according to their unique barcodes. High-quality clean tags were clustered into operational taxonomic units (OTUs) using USEARCH according to the QIIME quality-controlled process based on 97% sequence similarity. Further analysis was performed with representative OTUs using the Greengenes database with the RDP algorithm.

### 2.5. Determination of SCFA Concentrations

Fresh fecal pellets were mixed with deionized ice-cold water intermittently on a vortex mixer for 2 min. After maintained on ice for 20 min, the samples were centrifuged at 4800 g for 20 min at 4°C. The supernatant was collected and analyzed by injection onto the chromatographic system as previously described [15].

### 2.6. Western Blotting Analysis

Total proteins extracted from brain samples were used for western blotting determination as previously described [16]. Briefly, 20 *μ*g protein per lane was separated by SDS-PAGE and then blotted onto PVDF membranes. After blocked with skim milk, the membrane was incubated with primary antibodies against AMPK, phosphorylated AMPK, NF*κ*B p65, and phosphorylated NF*κ*B p65 (Cell Signaling, Danvers, MA, USA) overnight at 4°C. Then, after incubating with the secondary antibody at 22 ± 2°C for 1 h, the membrane was detected using the EZ-ECL (Biological Industries, Cromwell, CT, USA).

### 2.7. Statistical Analysis

Statistical analysis was performed using the *t*-test or one-way ANOVA with the data statistics software SPSS 18.0. *P* < 0.05 was considered statistically significant. All the measurement data are expressed as the means ± standard error (SEM).

## 3. Results

### 3.1. Serine and Glycine Deficiency Exacerbated the Accumulation of AGEs and MDA in D-Galactose-Treated Mice

As shown in Table 1, no significant difference was observed in AGE content in serum and MDA content in both serum and brain between the mice in the Control group and the SGD group. However, AGE content in serum and MDA content in both serum and brain were significantly higher in the D-gal group than those in the Control group. Moreover, the mice in the D-SGD group had the highest contents of AGEs and MDA, which were significantly higher than the mice in the other three groups.

### 3.2. Serine and Glycine Deficiency Exacerbated Inflammation and Oxidative Stress in D-Galactose-Treated Mice

As shown in Table 2, no significant difference in TNF-*α*, IL-1*β*, and IL-6 contents in serum was observed between the mice in the Control group and the SGD group. However, the contents of these inflammatory cytokines were significantly higher in the D-gal group than those in the Control group. Moreover, the mice in the D-SGD group had significantly higher concentration of these inflammatory cytokines than those in the other three groups.

As shown in Table 3, no significant difference was observed in serum activities of SOD, CAT, GSH-Px, and GSH content between the mice in the Control group and the SGD group. However, activities of these antioxidant enzymes and GSH content were significantly lower in the D-gal group than those in the Control group. Moreover, the mice in the D-SGD group had significantly lower activities of antioxidant enzymes and lower GSH content than those in the other three groups.

**Figure 1:**
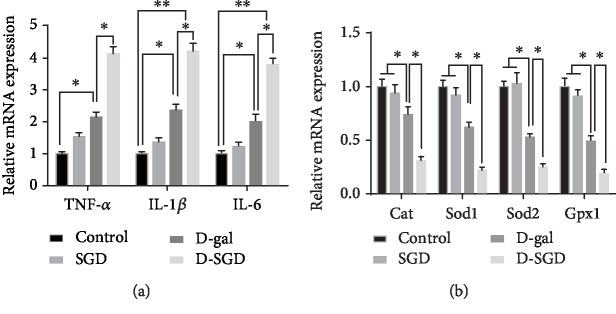
Effects of serine deficiency on the mRNA expression of genes encoding inflammatory cytokines and antioxidant enzymes in D-galactose-treated mice. (a) Relative mRNA expression of *TNF-α*, *IL-1β*, and *IL-6* in the brain; (b) relative mRNA expression of *Cat*, *Sod1*, *Sod2*, and *Gpx1* in the brain. Data were expressed as mean ± SEM, *n* = 8. ^∗^*P* < 0.05, ^∗∗^*P* < 0.01.

As shown in Figure 1, serine and glycine deficiency did not affect the mRNA expression of *TNF-α*, *IL-1β*, *IL-6*, *Cat*, *Sod1*, *Sod2*, and *Gpx1* in the brain of mice without D-galactose treatment. However, under D-galactose treatment, the mRNA expression of *TNF-α*, *IL-1β*, and *IL-6* was significantly increased, while the mRNA expression of *Cat*, *Sod1*, *Sod2*, and *Gpx1* was significantly decreased in mice fed SGD diet when compared with the mice fed the basal diet.

### 3.3. Serine and Glycine Deficiency Exacerbated Structure Change of Microbiota and Decreased SCFA Levels in D-Galactose-Treated Mice

As shown in Table 4, among the bacterial *α*-diversity, there was no significant difference between the groups regarding Simpson index. However, the Shannon H and observed species indexes decreased significantly in the D-gal and SGD groups when compared with the Control group. These two *α*-diversity indexes were further decreased in the D-SGD group when compared with either the D-gal group or the SGD group.

**Figure 2:**
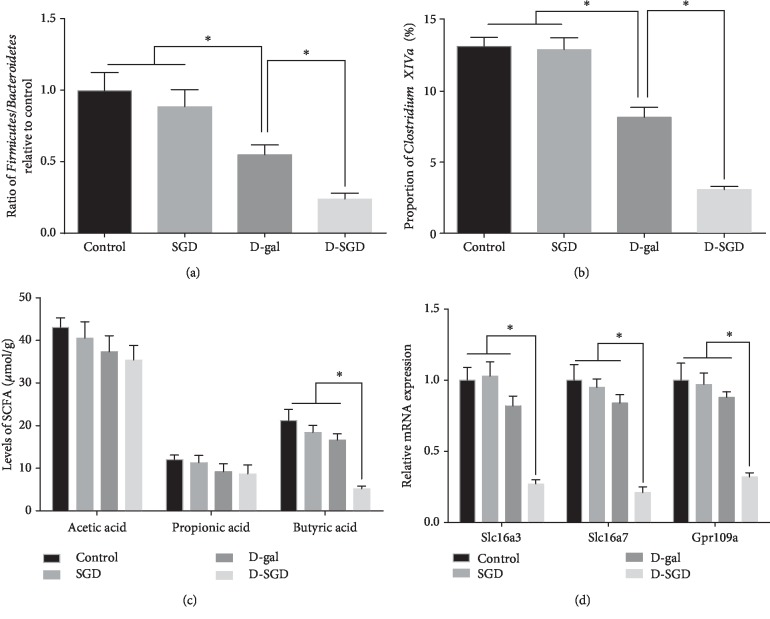
Serine deficiency exacerbated structure change of microbiota and decreased SCFA levels in D-galactose-treated mice. (a) Ratio of *Firmicutes*/*Bacteroidetes* in fecal microbiota; (b) proportion of *Clostridium XIVa*; (c) levels of short-chain fatty acids (SCFA) in feces; (d) relative mRNA expression of *Slc16a3*, *Slc16a7*, and *Gpr109a* in the brain. Data were expressed as mean ± SEM, *n* = 8. ^∗^*P* < 0.05.

Furthermore, we compared distinct community profiles at different taxonomic levels among the groups. At the phylum level, the results showed that the ratio of *Firmicutes*/*Bacteroidetes* was not significantly affected by serine and glycine deficiency (Figure 2(a)). However, under D-galactose treatment, the ratio of *Firmicutes*/*Bacteroidetes* was significantly decreased in mice fed SGD diet when compared with the mice fed the basal diet. At the genus level, D-galactose injection significantly decreased the proportion of *Clostridium XIVa* (Figure 2(b)). Moreover, serine and glycine deficiency exacerbated the decease of the proportion of *Clostridium XIVa* resulted from D-galactose injection.

Then, we determined the levels of SCFA in fresh feces. As shown in Figure 2(c), among the treatment groups, there were no significant difference regarding the levels of acetic acid and propionic acid. However, although serine and glycine deficiency or D-galactose injection alone did not change the level of butyric acid, SGD diet in combination of D-galactose injection significantly decreased the level of butyric acid. Additionally, mRNA expression of SCFA transporters and receptor in the brain was determined. The results showed that mRNA expression of *Slc16a3*, *Slc16a7*, and *Gpr109a* was significantly decreased in the D-SGD groups when compared with the other three treatment groups (Figure 2(d)).

### 3.4. Serine and Glycine Deficiency Inhibited the AMPK-NF*κ*B Signaling Pathway in D-Galactose-Treated Mice

**Figure 3:**
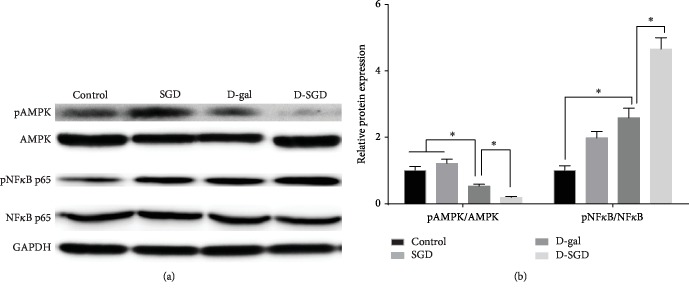
Effects of serine deficiency on AMPK and NF*κ*B expression in D-galactose-treated mice. (a) Protein expression of AMPK, pAMPK, NF*κ*B p65, and pNF*κ*B p65; (b) Relative expression of pAMPK and pNF*κ*B p65 according to the results of (a). Data were expressed as mean ± SEM, *n* = 3. ^∗^*P* < 0.05.

As shown in Figure 3, no significant difference was observed in the protein expression of pAMPK and pNF*κ*B p65 between the Control group and the SGD group. However, the protein expression of pAMPK was significantly decreased while pNF*κ*B p65 were significantly increased in the D-gal group when compared with the Control group. Furthermore, the changes of expression of these two phosphorylated proteins were much more significant in the D-SGD group when compared with those in the D-gal group.

## 4. Discussion

Chronic D-galactose exposure is a common model for exploring the mechanisms of age-related diseases. In this model, the formation of AGEs is considered as a trigger for the initiation of age-related diseases [17]. In the present study, we detected increased AGE content in the circulation of mice treated with D-galactose, suggesting that the aging model was successfully built and the mice were under the condition of high risks of developing age-related diseases. Accumulated AGEs are often associated with increased oxidative stress and inflammation as abnormal D-galactose metabolism results in the overproduction of reactive oxygen species (ROS) [18, 19]. ROS reacts with lipids to produce more stable products such as malondialdehyde (MDA), which further damage nucleic acids and proteins in the brain and lead to inflammatory aging [20]. In the present study, we found increased MDA content in both serum and brain, as well as increased serum contents of inflammatory cytokines and decreased activities of antioxidant enzymes in mice after chronic treatment with D-galactose. Moreover, genes encoding inflammatory cytokines were increased while genes encoding antioxidant enzymes were decreased in the brain. These results further confirmed the oxidative and inflammatory responses by D-galactose exposure, which were in line with previous results [17, 21]. Serine is a major substrate for the synthesis of GSH and NADPH [22, 23], suggesting that its availability plays a key role in the support of the antioxidant systems. Serine deficiency was previously proved to exacerbate oxidative stress [12]. In accordance with this report, our results showed that serine deficiency also exacerbates oxidative and inflammatory responses in the brain of aging mice. Our results suggested that the availability of serine might be important for aging individuals although whether extra serine supplementation might be beneficial for the prevention of age-related diseases remains to be explored.

Generally, the major species of the microbiota was proved to remain unchanged in older individuals, except that there is a reduced microbiota diversity [3]. We found that Shannon H and observed species were significantly decreased in mice treated with D-galactose, suggesting a reduction of richness of gut microbiota [24]. Moreover, we observed a significant decrease of the *Firmicutes* to *Bacteroidetes* ratio, which is considered as the most remarkable changes in older individuals [25]. Surprisingly, the *α*-diversity of microbiota and the *Firmicutes* to *Bacteroidetes* ratio were further decreased when the mice were fed a serine- and glycine-deficient diet. These results indicated that serine deficiency affected the microbiota composition in older individuals.

Recently, increasing evidences suggested that the microbiota could regulate brain function through the microbiota-gut-brain axis which enables microbes to communicate with the brain [26]. We found a decrease of the proportion of *Clostridium XIVa* at the genus level in mice treated with D-galactose, and its proportion was further decreased when the mice were fed a serine- and glycine-deficient diet. *Clostridium XIVa* is one of the producers of butyric acids [27]. In accordance with this, we determined the content of SCFAs in the feces and found a decrease of butyric acid in these mice. SCFAs were a bunch of microbial metabolites which could mediate the communication between microbes and the brain [28]. They can reach the brain since they are able to cross the blood-brain barrier according to the results of a cell culture model [29]. In association with the decreased content of butyric acid in the feces, we also found that the expression of its transporters and receptor was decreased in mice treated with D-galactose and a serine- and glycine-deficient diet. These results suggested that serine deficiency may reduce the communications between the gut microbiota and the brain through decreasing the production of butyric acid. SCFAs could enter the mitochondrial citric acid cycle and then generate energy [30]. Since AMPK is the key energy sensor, the AMPK-NF*κ*B signaling pathway was supposed to mediate the effects of SCFAs, as SCFA could activate AMPK and inhibit NF*κ*B activation [31]. Our results showed that D-galactose exposure inhibited AMPK phosphorylation while increased NF-*κ*B phosphorylation in the brain. Moreover, these changes were much more significant in mice treated with both D-galactose and a serine- and glycine-deficient diet. These results suggested that the AMPK-NF*κ*B signaling pathway also play a role in modulating oxidative and inflammatory responses which was exacerbated by serine deficiency.

In conclusion, our results showed that serine deficiency exacerbated oxidative and inflammatory status in an aging mouse model induced by chronic D-galactose treatment. Furthermore, our results suggested that the interactions between the microbiota and the brain through microbiota-gut-brain axis might mediate the abovementioned changes. To be specific, serine deficiency decreased the gut butyrate producer *Clostridium XIVa* and resulted in decreased production of butyric acid. Subsequently, the decreased butyric acid affected the phosphorylation of AMPK which promotes NF*κ*B activation and the accumulation of its downstream targets.

## Figures and Tables

**Table 1:** Effects of serine deficiency on levels of AGEs and MDA in D-galactose-treated mice.

Index	Control	SGD	D-gal	D-SGD
AGEs (pg/mL)	304 ± 25^a^	350 ± 30^a^	562 ± 37^b^	693 ± 32^c^
MDA in serum (nmol/mL)	2.05 ± 0.17^a^	2.49 ± 0.26^a^	3.79 ± 0.33^b^	5.86 ± 0.57^c^
MDA in brain (nmol/mg protein)	2.42 ± 0.28^a^	3.05 ± 0.41^a^	5.44 ± 0.53^b^	7.13 ± 0.49^c^

^a,b,c^Mean values within a row with unlike superscript letters were significantly different (*P* < 0.05). *n* = 8.

**Table 2:** Effects of serine deficiency on levels of serum inflammatory cytokines in D-galactose-treated mice.

Index	Control	SGD	D-gal	D-SGD
TNF-*α* (pg/mL)	114.2 ± 7.9^a^	120.3 ± 6.8^a^	143.5 ± 11.2^b^	177.8 ± 14.4^c^
IL-1*β* (pg/mL)	57.3 ± 3.6^a^	60.7 ± 4.1^a^	87.8 ± 3.1^b^	112.4 ± 6.3^c^
IL-6 (pg/mL)	145.3 ± 12.3^a^	158.9 ± 13.6^a^	209.4 ± 19.4^b^	283.8 ± 20.1^c^

^a,b,c^Mean values within a row with unlike superscript letters were significantly different (*P* < 0.05). *n* = 8.

**Table 3:** Effects of serine deficiency on activities of antioxidant enzymes and GSH concentration in the serum of D-galactose-induced aging mice.

Index	Control	SGD	D-gal	D-SGD
SOD (U/mL)	34.1 ± 2.3^a^	32.5 ± 2.1^a^	24.9 ± 1.5^b^	18.4 ± 1.3^c^
CAT (U/L)	23.1 ± 2.3^a^	19.8 ± 2.5^a^	14.3 ± 2.0^b^	7.5 ± 1.7^c^
GSH-Px (U/mL)	31.0 ± 3.7^a^	26.1 ± 2.3^a^	19.3 ± 1.6^b^	7.9 ± 1.9^c^
GSH (nmol/mL)	615.3 ± 36.3^a^	552.2 ± 29.4^a^	421.4 ± 25.0^b^	228.2 ± 27.6^c^

^a,b,c^Mean values within a row with unlike superscript letters were significantly different (*P* < 0.05). *n* = 8.

**Table 4:** Effects of serine deficiency on *α*-diversity indexes of microbiota in D-galactose-induced aging mice.

Index	Control	SGD	D-gal	D-SGD
Shannon H	6.72 ± 0.52^a^	5.13 ± 0.39^b^	4.62 ± 0.57^b^	3.09 ± 0.30^c^
Simpson	0.84 ± 0.11	0.75 ± 0.09	0.71 ± 0.10	0.66 ± 0.18
Observed species	317.3 ± 25.8^a^	269.9 ± 27.4^b^	248.1 ± 19.3^b^	201.7 ± 22.3^c^

^a,b,c^Mean values within a row with unlike superscript letters were significantly different (*P* < 0.05). *n* = 8.

## Data Availability

The data used to support the findings of this study are included with the article.

## Supplementary Materials

Supplementary Materials Supplementary Table 1: sequences of primers used for RT-qPCR in the experiments. Supplementary Table 2: components of the control diet and serine- and glycine-deficient (SGD) diet used in the experiments. These two diets were purchased from Research Diets (New Brunswick, NJ, USA).

## References

[1] Poon H. F., Calabrese V., Scapagnini G., Butterfield D. A. (2004). Free radicals: key to brain aging and heme oxygenase as a cellular response to oxidative stress. *The Journals of Gerontology. Series A, Biological Sciences and Medical Sciences*.

[2] Spychala M. S., Venna V. R., Jandzinski M. (2018). Age-related changes in the gut microbiota influence systemic inflammation and stroke outcome. *Annals of Neurology*.

[3] Dinan T. G., Cryan J. F. (2017). Gut instincts: microbiota as a key regulator of brain development, ageing and neurodegeneration. *The Journal of Physiology*.

[4] Garcia-Pena C., Alvarez-Cisneros T., Quiroz-Baez R., Friedland R. P. (2017). Microbiota and aging. A review and commentary. *Archives of Medical Research*.

[5] Castrogiovanni P., Li Volti G., Sanfilippo C. (2018). Fasting and fast food diet play an opposite role in mice brain aging. *Molecular Neurobiology*.

[6] Jiang Q., Lu C., Sun T. (2019). Alterations of the brain proteome and gut microbiota in d-galactose-induced brain-aging mice with krill oil supplementation. *Journal of Agricultural and Food Chemistry*.

[7] He L., Long J., Zhou X., Liu Y., Li T., Wu X. (2020). Serine is required for the maintenance of redox balance and proliferation in the intestine under oxidative stress. *The FASEB Journal*.

[8] Zhou X., He L., Wu C., Zhang Y., Wu X., Yin Y. (2017). Serine alleviates oxidative stress via supporting glutathione synthesis and methionine cycle in mice. *Molecular Nutrition & Food Research*.

[9] Zhou X., He L., Zuo S. (2018). Serine prevented high-fat diet-induced oxidative stress by activating AMPK and epigenetically modulating the expression of glutathione synthesis-related genes. *Biochimica et Biophysica Acta - Molecular Basis of Disease*.

[10] Zhou X., Zhang Y., He L. (2017). Serine prevents LPS-induced intestinal inflammation and barrier damage via p53-dependent glutathione synthesis and AMPK activation. *Journal of Functional Foods*.

[11] Zhang H., Hua R., Zhang B., Zhang X., Yang H., Zhou X. (2018). Serine alleviates dextran sulfate sodium-induced colitis and regulates the gut microbiota in mice. *Frontiers in Microbiology*.

[12] He L., Zhang H., Zhou X. (2018). Weanling offspring of dams maintained on serine-deficient diet are vulnerable to oxidative stress. *Oxidative Medicine and Cellular Longevity*.

[13] Guan G., Ding S., Yin Y., Duraipandiyan V., Al-Dhabi N. A., Liu G. (2019). Macleaya cordata extract alleviated oxidative stress and altered innate immune response in mice challenged with enterotoxigenic Escherichia coli. *Science China Life Sciences*.

[14] Wang K., Jin X., You M. (2017). Dietary propolis ameliorates dextran sulfate sodium-induced colitis and modulates the gut microbiota in rats fed a Western diet. *Nutrients*.

[15] Sha J. Y., Zhou Y. D., Yang J. Y. (2019). Maltol (3-hydroxy-2-methyl-4-pyrone) slows d-galactose-induced brain aging process by damping the Nrf2/HO-1-mediated oxidative stress in mice. *Journal of Agricultural and Food Chemistry*.

[16] Liu G., Chen S., Guan G. (2016). Chitosan modulates inflammatory responses in rats infected with enterotoxigenic Escherichia coli. *Mediators of Inflammation*.

[17] Zhang X., Jin C., Li Y., Guan S., Han F., Zhang S. (2013). Catalpol improves cholinergic function and reduces inflammatory cytokines in the senescent mice induced by D-galactose. *Food and Chemical Toxicology*.

[18] Cannizzo E. S., Clement C. C., Sahu R., Follo C., Santambrogio L. (2011). Oxidative stress, inflamm-aging and immunosenescence. *Journal of Proteomics*.

[19] Semba R. D., Nicklett E. J., Ferrucci L. (2010). Does accumulation of advanced glycation end products contribute to the aging phenotype?. *The Journals of Gerontology Series A: Biological Sciences and Medical Sciences*.

[20] Gawel S., Wardas M., Niedworok E., Wardas P. (2004). Malondialdehyde (MDA) as a lipid peroxidation marker. *Wiadomości Lekarskie*.

[21] Li B., Evivie S. E., Lu J. (2018). Lactobacillus helveticusKLDS1.8701 alleviatesd-galactose-induced aging by regulating Nrf-2 and gut microbiota in mice. *Food & Function*.

[22] Maddocks O. D. K., Labuschagne C. F., Adams P. D., Vousden K. H. (2016). Serine metabolism supports the methionine cycle and DNA/RNA methylation through de novo ATP synthesis in cancer cells. *Molecular Cell*.

[23] Zhou X., Zhang H., He L., Wu X., Yin Y. (2018). Long-term l-serine administration reduces food intake and improves oxidative stress and Sirt1/NF*κ*B signaling in the hypothalamus of aging mice. *Frontiers in Endocrinology*.

[24] Guan G., Wang H., Chen S. (2016). Dietary chitosan supplementation increases microbial diversity and attenuates the severity of Citrobacter rodentium infection in mice. *Mediators of Inflammation*.

[25] Saraswati S., Sitaraman R. (2014). Aging and the human gut microbiota-from correlation to causality. *Frontiers in Microbiology*.

[26] Foster J. A., Lyte M., Meyer E., Cryan J. F. (2016). Gut Microbiota and Brain Function: An Evolving Field in Neuroscience: Table 1. *International Journal of Neuropsychopharmacology*.

[27] Vital M., Karch A., Pieper D. H. (2017). Colonic butyrate-producing communities in humans: an overview using omics data. *mSystems*.

[28] Dalile B., Van Oudenhove L., Vervliet B., Verbeke K. (2019). The role of short-chain fatty acids in microbiota-gut-brain communication. *Nature Reviews Gastroenterology & Hepatology*.

[29] Mitchell R. W., On N. H., del Bigio M. R., Miller D. W., Hatch G. M. (2011). Fatty acid transport protein expression in human brain and potential role in fatty acid transport across human brain microvessel endothelial cells. *Journal of Neurochemistry*.

[30] Schonfeld P., Wojtczak L. (2016). Short- and medium-chain fatty acids in energy metabolism: the cellular perspective. *Journal of Lipid Research*.

[31] Clark A., Mach N. (2017). The crosstalk between the gut microbiota and mitochondria during exercise. *Frontiers in Physiology*.

