# Supplementary material for: Serine Deficiency Exacerbates Inflammation and Oxidative Stress via Microbiota-Gut-Brain Axis in D-Galactose-Induced Aging Mice
Source: Mediators Inflamm. 2020 Mar 2;2020:5821428. doi: 10.1155/2020/5821428 (PMC7071807; doi:10.1155/2020/5821428)
Supplement: Supplementary Materials — Supplementary Table 1: sequences of primers used for RT-qPCR in the experiments. Supplementary Table 2: components of the control diet and serine- and glycine-deficient (SGD) diet used in the experiments. These two diets were purchased from Research Diets (New Brunswick, NJ, USA). [file 5821428.f1.docx]

**Supplementary Table 1** Sequences of primers used for RT-qPCR in the experiments.

**Supplementary Table 2** Components of the control diet, and serine and glycine deficient (SGD) diet used in the experiments. These two diets were purchased from Research Diets (New Brunswick, NJ, USA).

**Supplementary Table 1** Primer sequences for RT-qPCR.

| Gene | 5’-3’ Primer sequence |
| --- | --- |
| Cat | F: CAAGATTGCCTTCTCCGGGT |
|  | R: GACCCCGCGGTCATGATATT |
| Sod1 | F: ATTGGCCGTACAATGGTGGT |
|  | R: ATCCCAATCACTCCACAGGC |
| Sod2 | F: GTAGGGCCTGTCCGATGATG |
|  | R: CGCTACTGAGAAAGGTGCCA |
| Gpx1 | F: ACAGTCCACCGTGTATGCCT |
|  | R: ACCAGGTCGGACGTACTTGA |
| IL-1β | F: TGCCACCTTTTGACAGTGATG |
|  | R: AAGGTCCACGGGAAAGACAC |
| IL-6 | F: CCTCTCTGCAAGAGACTTCCAT |
|  | R: AGTCTCCTCTCCGGACTTGT |
| TNF-α | F: ATGAGAAGTTCCCAAATGGC |
|  | R: CTCCACTTGGTGGTTTGCTA |
| β-actin | F: TGTCCACCTTCCAGCAGATGT |
|  | R: AGCTCAGTAACAGTCCGCCTAGA |

**Supplementary table 2** Diet components.

| Components | Control diet, gm | SGD diet, gm |
| --- | --- | --- |
| L-Arginine | 10 | 10 |
| L-Histidine-HCl-H_2_O | 6 | 6 |
| L-Isoleucine | 8 | 8 |
| L-Leucine | 12 | 12 |
| L-Lysine-HCl | 14 | 14 |
| L-Methionine | 6 | 6 |
| L-Phenylalanine | 8 | 8 |
| L-Threonine | 8 | 8 |
| L-Tryptophan | 2 | 2 |
| L-Valine | 8 | 8 |
| L-Alanine | 10 | 10 |
| L-Asparagine-H_2_O | 5 | 5 |
| L-Aspartate | 10 | 10 |
| L-Cystine | 4 | 4 |
| L-Glutamic Acid | 30 | 30 |
| L-Glutamine | 5 | 5 |
| L-Glycine | 10 | 0 |
| L-Proline | 5 | 5 |
| L-Serine | 5 | 0 |
| L-Tyrosine | 4 | 4 |
| Corn Starch | 550.5 | 565.5 |
| Maltodextrin 10 | 125 | 125 |
| Cellulose | 50 | 50 |
| Corn Oil | 50 | 50 |
| Mineral Mix S10001 | 35 | 35 |
| Sodium Bicarbonate | 7.5 | 7.5 |
| Vitamin Mix V10001 | 10 | 10 |
| Choline Bitrartrate | 2 | 2 |
| Total | 1000 | 1000 |
